# Comparing the job satisfaction and intention to leave of different categories of health workers in Tanzania, Malawi, and South Africa

**DOI:** 10.3402/gha.v6i0.19287

**Published:** 2013-01-24

**Authors:** Duane Blaauw, Prudence Ditlopo, Fresier Maseko, Maureen Chirwa, Aziza Mwisongo, Posy Bidwell, Steve Thomas, Charles Normand

**Affiliations:** 1 Centre for Health Policy, School of Public Health, Faculty of Health Sciences, University of the Witwatersrand, Johannesburg, South Africa; 2 Department of Community Health, College of Medicine, University of Malawi, Blantyre, Malawi; 3 National Institute for Medical Research, Dar es Salaam, Tanzania; 4 Centre for Global Health, Trinity College, University of Dublin, Dublin, Ireland

**Keywords:** health worker, job satisfaction, intention to leave, Tanzania, Malawi, South Africa

## Abstract

**Background:**

Job satisfaction is an important determinant of health worker motivation, retention, and performance, all of which are critical to improving the functioning of health systems in low- and middle-income countries. A number of small-scale surveys have measured the job satisfaction and intention to leave of individual health worker cadres in different settings, but there are few multi-country and multi-cadre comparative studies.

**Objective:**

The objective of this study was to compare the job satisfaction and intention to leave of different categories of health workers in Tanzania, Malawi, and South Africa.

**Methods:**

We undertook a cross-sectional survey of a stratified cluster sample of 2,220 health workers, 564 from Tanzania, 939 from Malawi, and 717 from South Africa. Participants completed a self-administered questionnaire, which included demographic information, a 10-item job satisfaction scale, and one question on intention to leave. Multiple regression was used to identify significant predictors of job satisfaction and intention to leave.

**Results:**

There were statistically significant differences in job satisfaction and intention to leave between the three countries. Approximately 52.1% of health workers in South Africa were satisfied with their jobs compared to 71% from Malawi and 82.6% from Tanzania (χ^2^=140.3, *p*<0.001). 18.8% of health workers in Tanzania and 26.5% in Malawi indicated that they were actively seeking employment elsewhere, compared to 41.4% in South Africa (χ^2^=83.5, *p*<0.001). The country differences were confirmed by multiple regression. The study also confirmed that job satisfaction is statistically related to intention to leave.

**Conclusions:**

We have shown differences in the levels of job satisfaction and intention to leave between different groups of health workers from Tanzania, Malawi, and South Africa. Our results caution against generalising about the effectiveness of interventions in different contexts and highlight the need for less standardised and more targeted HRH strategies than has been practised to date.

Inadequate human resources are a major constraint to improving global health (1, 2). The insufficient numbers, skill imbalances, maldistribution, low motivation, and poor performance of health workers in low-and middle-income countries (LMICs) compromise the delivery and expansion of priority health programmes (3, 4). Addressing the human resources for health (HRH) crisis is now a major component of health systems strengthening initiatives in LMICs.

The job satisfaction of health workers has become an important topic of HRH research (5, 6). Job satisfaction is the degree to which employees have a positive affective orientation towards employment by an organisation (7). Job satisfaction has been conceptualised both *globally* (general satisfaction with a job) and *dimensionally* (satisfaction with specific dimensions of a job such as remuneration, promotion, and relationships with colleagues) (7). Job satisfaction is of interest because it is an important determinant of the performance of health professionals. Job satisfaction has been linked to health worker motivation (8), stress (9, 10), burnout (11), absenteeism (12, 13), intention to leave (14–16), and turnover (17, 18). Intention to leave refers to an employee's expressed intention of leaving their current job in the near future (19) and is used as a proxy indicator of turnover in cross-sectional surveys, which are not able to measure turnover directly (15).

The job satisfaction of health professionals has been shown to be influenced by a range of individual and organisational factors (5, 20–23), including personality, the work itself, work organisation, remuneration, workload, interactions with colleagues, training opportunities, recognition, and leadership styles. Importantly, job satisfaction depends not only on the characteristics of a job but also on employees’ expectations of what their job should provide (5).

Job satisfaction studies have now been conducted in a number of different countries with different categories of health workers using a range of tools. The vast majority of research on health worker job satisfaction has been conducted in high-and upper middle-income countries (5, 6), and there are only very few studies from lower income countries (24–32). Nurses are, by far, the most researched group (5), but there are also a number of studies with doctors, dentists and pharmacists (6, 33–35). Research on other allied health workers has been less common (36). The job satisfaction of nurse practitioners in high-income countries has been investigated (37, 38) but we were only able to find one study relating to the job-satisfaction of mid-level workers in Africa (39).

Most job satisfaction data derives from small-scale surveys conducted with a single category of health worker from one country. There are few job satisfaction studies formally comparing different countries (25, 40–45) or different health worker cadres (46–50), and we were not able to find any studies that do both. Comparative research would help elucidate the specific needs of different health workers in specific contexts and inform the design of more effective HRH interventions (51).

Intention to leave is an intervening variable between job satisfaction and actual turnover (19) and is therefore affected by similar individual and organisational factors. The literature on intention to leave among health workers suffers from the same limitations highlighted above. There are very few studies from low-income and lower middle-income countries (30) and only a handful from upper middle-income countries (52–56). We were only able to find one relevant comparative study, a comparison of organisational commitment in Malaysian and English nurses (45).

The objective of this study was to measure and compare the job satisfaction and intention to leave of different categories of health workers in Tanzania, Malawi, and South Africa.

## Methods

### Study context

This research was part of a broader study, *The Motivation Project*, that investigated issues of motivation and retention of health workers in Tanzania, Malawi, and South Africa (57). Table 1 compares some key demographic, health expenditure, health status, and HRH indicators from the three countries. These three countries were selected for the larger study because of differences in health professional cadres, regulatory environments, health labour market, resource availability, and recent HRH policy interventions. Tanzania and Malawi are low-income countries with large rural populations and high levels of poverty, whereas South Africa is a middle-income country that is more urbanised. South Africa has higher health expenditure per capita than Tanzania and Malawi, and significantly more health workers, although the health outcomes of the three countries are similar. The HRH crisis in Malawi and Tanzania is particularly severe, even in comparison to other countries in sub-Saharan Africa. Both countries have very low numbers of health professionals, depend on mid-level clinical officers and nurses to provide health care services, and have documented problems with health worker motivation (59, 60). The South African national HRH statistics are better in comparison, but obscure significant problems in retaining and motivating health workers in the public health sector and rural areas (61). All three countries have on-going HRH interventions to address these problems which would be informed by better information on job satisfaction.

**Table 1:** Comparison of key indicators in study countries – 2010

Indicator	Tanzania	Malawi	South Africa
Population (Million)	42.5	14.8	49.7
Population in rural areas	75%	81%	39%
GNP per capita (PPP)	$1,230	$830	$9,780
Population living on < $1 (PPP) per day	88.5%	73.9%	26.2%
Total expenditure on health (% GDP)	5.3%	9.9%	8.6%
Per capita total expenditure on health (PPP)	$22	$50	$819
Life expectancy at birth	53 yrs	53 yrs	53 yrs
IMR (per 1,000 live births)	67	65	48
MMR (per 100,000 live births)	950	1,100	400
HIV prevalence	6.2%	11.9%	18.1%
Number of nurses	9,440	3,896	184,459
Nurses per 10,000 population	2	3	41
Number of doctors	300	257	34,829
Doctors per 10,000 population	<0.5	<0.5	8

Source: WHO World Health Statistics (58).

### Data collection

As detailed below, multi-level stratified sampling strategies, customised for each country, were used to select a reasonably representative sample of health workers. We aimed to include approximately 150 respondents from each of the main cadres of health workers in each country to allow comparisons between groups and countries. The study included health workers from both public sector and non-governmental health facilities but not from the private-for-profit sector.

The study was based in three regions of Tanzania. Two districts were randomly selected from each region and data were then collected from the regional and district hospitals, as well as two health centres and three dispensaries selected at random from each district. In each of the selected facilities, random samples of health workers from the predetermined categories present on the day of the study were selected: 100 participants from each regional hospital, 40 from each district hospital, 8 from each health centre, and 3 from each dispensary. In Malawi, 11 of the 27 districts were selected at random, and all health workers from the predetermined categories in the selected districts were included in the sample. In South Africa, the study was based in two provinces (one urban and one rural) selected for the broader study. A random sample of five district hospitals and one referral hospital were randomly selected from each province, and then four wards in each district hospital and six wards in each referral hospital were randomly selected. The survey sample included all doctors and six nurses chosen at random from personnel lists in the selected wards.

To enable comparisons, we intended to include the most important health worker cadres in each country. The targeted groups in Tanzania and Malawi were nursing auxiliaries (1 year training), enrolled nurses (2 years), registered nurses (4 years), clinical officers (3 years), and allied health staff. Medical officers were included if they were present in the sampled facilities at the time of the survey, but they make up a very small proportion of the health workforce in these two countries. Although South Africa has begun to train small numbers of mid-level workers (62), none had graduated by the time of the study, so the survey concentrated on nursing auxiliaries, enrolled nurses, registered nurses, and doctors (medical officers and specialists) in the study facilities.

The study was explained to the selected health workers and they were invited to participate. Those that agreed completed a self-administered questionnaire which included basic socio-demographic information, a 10-item job satisfaction scale, and one question on intention to leave (19). The job satisfaction scale was derived from Bennett et al. (63) who undertook an extensive review of organisational measurement scales and validated their tools in studies in Georgia and Jordan (64). These tools have successfully been used in other African settings (65). Respondents were asked to indicate their agreement with statements about their satisfaction with their job in general and with nine different aspects of their jobs, using a five-point Likert scale from ‘strongly disagree’ to ‘strongly agree’. The job attributes covered both intrinsic and extrinsic job satisfaction (63) and focused on important aspects highlighted in our previous review (57). The question on intention to leave asked health workers how strongly they agreed with the statement that they were actively seeking other employment.

### Data analysis

Data from the questionnaires in each country was entered into an Epi-Info database and then transferred to Stata v12.0 (StataCorp, College Station, TX) for cleaning, merging, and analysis.

Statistical differences in socio-demographic characteristics between the three countries were evaluated by means of a Chi-squared test for categorical variables and analysis of variance (ANOVA) for numerical variables. Post-hoc analysis of the ANOVA results used the Bonferroni test to identify which differences between countries were significant. 95% confidence intervals (95% CI) were calculated using standard methods, including exact binomial intervals for proportions.

Two methods were used for the analysis of the Likert scale responses. Firstly, the answers were coded on a scale from 1 (strongly disagree) to 5 (strongly agree) and analysed numerically. Secondly, we calculated the proportion of respondents agreeing with each statement by combining those that ‘strongly agreed’ and those that ‘agreed’ with each statement. Differences between countries were tested using ANOVA of the numerical data.

We used principal component analysis (PCA) to construct a job satisfaction index combining all ten questions on job satisfaction. The reliability of this scale was evaluated by calculating Cronbach's alpha and by inspection of the results of the PCA.

Finally, to formally test for differences between the three countries while adjusting for other socio-demographic determinants, we used multiple linear regression for job satisfaction and multiple logistic regression for intention to leave. For job satisfaction, we used the job satisfaction index from the PCA. For intention to leave we used the Likert response recoded as a binary variable indicating those health workers who agreed that they were actively seeking alternative employment. A cut-off of 0.05 was used to determine statistical significance in all the statistical tests.

### Ethical considerations

National and international ethical standards were followed throughout the research. The study protocol was reviewed by the human research ethics committees of the National Institute for Medical Research in Tanzania, the University of the Witwatersrand in South Africa, the University of Malawi in Malawi, and Trinity College Dublin in Ireland. Prior to data collection, permission to conduct the research was also obtained from the relevant governmental and health authorities in each country. Written informed consent was obtained from all participants.

## Results

### Study participants

A total of 2,335 respondents were selected to participate in the survey. We excluded non-health workers and blank questionnaires from those that indicated they did not want to participate. This left a total of 2,221 questionnaires for analysis: 567 from Tanzania, 937 from Malawi, and 717 from South Africa, giving response rates for the study of 91.7%, 95.1, and 98.0%, respectively.

Table 2 summarises the basic demographic characteristics of the study sample. The health workers in this study were predominantly female, half were married, and most had children. The mean age of the participants was 38.2 years, and they had been working in their current jobs for an average of nearly 8 years. All of these variables showed statistically significant differences between the three study countries (Table 2). In particular, the Malawian sample had a higher proportion of males, was relatively younger, had fewer children, and had been in a post for a much shorter period of time, in comparison to study participants from South Africa and Tanzania.

**Table 2:** Characteristics of study participants

Variables	Total	Tanzania	Malawi	South Africa	*p*
N	2,221	567	937	717	
Sex					
% Female	62.5	68.2	47.9	77.0	<0.01 †
Age					
Mean±SD	38.2±11.4	41.0±9.4	35.9±12.5	39.3±10.5	<0.01 ‡
Marital status					
% Single	35.0	23.2	37.7	40.7	<0.01 †
% Married	54.7	66.6	52.0	48.8	
Any children					
% Yes	70.5	82.7	60.9	73.4	<0.01 †
Facility type					
% Clinic	21.0	14.2	41.3	0.0	<0.01 †
% Public hospital	72.3	85.8	42.7	100.0	
% Mission hospital	6.8	0.0	16.1	0.0	
Years in current post					
Mean±SD	7.7±8.5	12.1±9.4	4.0±5.3	9.3±9.3	<0.01 ‡

† Chi-squared test

‡ ANOVA.

Table 3 provides a breakdown of the participants by cadre. In Tanzania, nursing staff (medical attendants, nurse midwives, trained nurses) made up 61.6% of health workers, 18.3% were clinical officers or assistant medical officers, and there were only 25 (4.4%) doctors. The main categories in Malawi were nurse midwife technicians and enrolled nurses (45.2%), and medical assistants and clinical officers (30.4%). Reflecting their relative scarcity, registered nurses were only 10.4% of the sample, and there were only five medical officers in total in the sampled facilities. Allied health workers (therapists, radiographers, pharmacists, laboratory technologists) made up a small proportion of participants from Malawi and Tanzania. Finally, in South Africa, the sample was more or less evenly divided between nursing auxiliaries, registered nurses and doctors.

**Table 3:** Breakdown of health worker cadres in each country

	Tanzania		Malawi		South Africa		Total	
	
Category	N	%	n	%	n	%	n	%
Auxiliary/enrolled nurse	163	28.8	424	45.2	258	36.0	845	38.0
Registered nurse	186	32.8	97	10.4	270	37.7	553	24.9
Medical assistant/clinical officer	104	18.3	285	30.4			389	17.5
Medical officer/specialist	25	4.4	5	0.5	189	26.3	219	9.9
Allied health workers	89	15.7	126	13.5			215	9.7
Total	567	100.0	937	100.0	717	100.0	2,221	100.0

### Job satisfaction and intention to leave

The results of the job satisfaction survey by country are summarised in Table 4. Overall, 82.3% [95% CI: 78.9–85.4] of respondents in Tanzania were satisfied with their jobs, compared to 71.0% [95% CI: 68.0–73.9] in Malawi, and 52.1% [95% CI: 48.3–55.8] in South Africa. These differences were statistically significant (χ^2^=138.6, *p*<0.001). The mean scores for each question were compared to evaluate satisfaction with different job attributes. In all three countries, health workers were most satisfied with their job variety and the opportunity to fully utilise their abilities. The lowest mean satisfaction scores in all three countries were for the educational and training opportunities and the availability of drugs and equipment. Although the rankings were reasonably consistent, the actual scores differed significantly between the three countries (Table 4). For all job attributes, satisfaction was generally highest in Tanzania, intermediate in Malawi, and lowest in South Africa. Post-hoc analysis of the ANOVA results showed that differences between all three countries were significant for most of these results, except that the mean scores for satisfaction with manager handling of staff and the availability of drugs were not statistically different between Malawi and South Africa, and that Malawi and Tanzania did not have statistically different scores for the question about jobs allowing health workers to perform at high levels (Table 4).

**Table 4:** Satisfaction with job characteristics and intention to leave by country

	% Agreeing			Satisfaction score (Mean±SD)			Significance (ANOVA)	
			
Job attribute	Tanzania	Malawi	South Africa	Tanzania	Malawi	South Africa	Overall	Post-hoc comparison
In general, I am satisfied with this job	82.3	71.0	52.1	4.03±0.91	3.72±1.23	3.22±1.19	F=82.28, *p*<0.001	a, b, c
I feel that I am able to use my abilities to their full potential	88.9	78.2	62.7	4.22±0.83	3.94±1.1	3.56±1.15	F=64.1, *p*<0.001	a, b, c
I have a variety of duties, tasks and activities in my job	88.5	93.5	86.7	4.19±0.83	4.33±0.78	4.04±0.87	F=25.3, *p*<0.001	a, b, c
I find that my opinions are respected at work	72.9	63.1	48.0	3.76±0.97	3.49±1.13	3.22±1.1	F=39.64, *p*<0.001	a, b, c
I am satisfied with the recognition I get for the work that I do	77.2	51.2	38.8	3.8±0.93	3.16±1.26	2.98±1.17	F=87.1, *p*<0.001	a, b, c
I am satisfied with the personal relationship between my manager and myself	75.6	65.6	55.1	3.82±0.98	3.55±1.16	3.35±1.17	F=28.4, *p*<0.001	a, b, c
I am satisfied with the way my manager handles staff	62.8	46.8	43.2	3.53±1.07	3.11±1.21	3.02±1.22	F=32.9, *p*<0.001	b, c
I feel that my job conditions allow me to perform at high levels	60.0	55.9	43.3	3.47±1.18	3.35±1.25	3.03±1.2	F=22.8, *p*<0.001	a, c
I am satisfied with the availability of drugs and equipment	46.2	39.9	36.7	3.11±1.18	2.85±1.2	2.78±1.24	F=12.7, *p*<0.001	b, c
I am satisfied with the educational/training opportunities that I get	41.0	29.9	39.2	2.98±1.17	2.53±1.28	2.8±1.29	F=24.7, *p*<0.001	a, b, c
I am actively seeking other employment	18.8	26.5	41.4	2.42±1.13	2.64±1.31	3.05±1.34	F=39.9, *p*<0.001	a, b, c

Bonferroni post-hoc comparisons: a: Malawi and South Africa significantly different; b: Malawi and Tanzania significantly different; c: South Africa and Tanzania significantly different.

In terms of intending to leave their current job, only 18.8% [95% CI: 15.6–22.2] of health workers in Tanzania and 26.5% [95% CI: 23.7–29.5] in Malawi indicated that they were actively seeking employment elsewhere, compared to 41.4% [95% CI: 37.3–45.1] in South Africa (Table 4). These differences between the three countries were also statistically significant (χ^2^=83.5, *p*<0.001).

The scores for satisfaction with different job components correlated well with each other and with general job satisfaction. The Cronbach's alpha for the satisfaction scale derived from all 10 items was 0.829 (0.823, 0.808, and 0.838 in Tanzania, Malawi, and South Africa, respectively). The question on job variety had the lowest correlation with the scale although this was still 0.435 and its exclusion did not improve Cronbach's alpha significantly. The first component in the PCA explained 40.8% of the total variance (39.6%, 37.5, and 42.4% in Tanzania, Malawi, and South Africa, respectively). The loadings indicated that an index derived from the first component was close to an average of all ten items (results not shown) and the correlation between a simple additive sum of the ten items and the first component was 0.997 (*p*<0.001). The job satisfaction index obtained from the first component of the PCA was used in subsequent regression analyses.

### Determinants of job satisfaction and intention to leave

Table 5 shows the results of the multiple regression used to evaluate differences in job satisfaction between the three countries while adjusting for differences in other socio-demographic determinants (Table 2). The model was statistically significant (*p*<0.001) but only explained 10.7% of the total variation in job satisfaction. Female health workers were less satisfied than males but this difference was not significant in the multiple regression. Job satisfaction was significantly higher in the age group over 50 years of age compared to those under 30 years. Workers in public hospitals were significantly less satisfied than workers in clinics and health centres, they were also less satisfied than workers in mission hospitals but this difference was not statistically significant (*p*=0.440). Differences between different health worker cadres were also not significant. However, the differences between countries persisted in the multiple regression after adjusting for other available predictors. Job satisfaction in South Africa was significantly lower than Malawi, while Tanzania scored significantly higher than Malawi.

**Table 5:** Multiple linear regression of predictors of job satisfaction

Variable	Coefficient	95% CI	*p*
Constant	−0.215	[−0.686; 0.256]	0.371
Female	−0.146	[−0.377; 0.086]	0.217
Age group			
< 30 years	–	–	–
30–50 years	0.177	[−0.081; 0.435]	0.178
> 50 years	0.751	[0.409; 1.093]	<0.001
Marital status			
Single	–	–	–
Married/living together	−0.015	[−0.249; 0.220]	0.901
Separated/divorced/widowed	0.405	[0.049; 0.761]	0.026
Number of children	0.005	[−0.256: 0.266]	0.970
Facility type			
Clinic/health centre	–	–	–
Public hospital	−0.440	[−0.693; −0.187]	0.001
Mission hospital	−0.180	[−0.567; 0.208]	0.363
Years in current post	0.002	[−0.011: 0.016]	0.730
Health worker cadre			
Auxiliary/enrolled nurse	–	–	–
Registered nurse	−0.087	[−0.328; 0.153]	0.476
Medical assistant/clinical officer	−0.219	[−0.514; 0.075]	0.144
Medical officer/specialist	0.062	[−0.317; 0.442]	0.748
Allied health workers	−0.021	[−0.367; 0.325]	0.905
Country			
Malawi	–	–	–
South Africa	−0.461	[−0.76; −0.161]	0.003
Tanzania	0.923	[0.657; 1.189]	<0.001

F=14.9, *p*<0.001, R^2^=0.107.

Table 6 shows that the multiple regression model of predictors of intention to leave. Gender, marital status, type of facility and health worker category were not significantly associated with actively seeking alternative employment. However, intention to leave decreased significantly with age – the odds of leaving in the over 50 age group was half that of those under 30 years. Intention to leave was statistically higher in South Africa than in Malawi (*p*<0.001) and in Tanzania (*p*<0.001), with South African health workers more than twice as likely to report on intending to leave than those from Malawi and Tanzania, but there was no significant difference between Malawi and Tanzania (*p*=0.871). Intention to leave was negatively correlated with job satisfaction in the multiple regression, so that health workers with higher job satisfaction indices were significantly less likely to want to leave their jobs.

**Table 6:** Multiple logistic regression of predictors of intention to leave

Variable	OR	95% CI	*p*
Constant	0.420	[0.239; 0.737]	0.003
Female	0.954	[0.722; 1.26]	0.741
Age group			
< 30 years	–	–	–
30–50 years	0.726	[0.535; 0.985]	0.039
> 50 years	0.495	[0.318; 0.770]	0.002
Marital status			
Single	–	–	–
Married/living together	0.838	[0.634; 1.107]	0.214
Separated/divorced/widowed	0.847	[0.537; 1.336]	0.475
Any children	1.244	[0.91; 1.701]	0.171
Facility type			
Clinic/health centre	–	–	–
Public hospital	0.895	[0.650; 1.231]	0.493
Mission hospital	1.085	[0.675; 1.743]	0.738
Years worked at this facility	0.989	[0.971; 1.007]	0.224
Health worker cadre			
Auxiliary/enrolled nurse	–	–	–
Registered nurse	1.088	[0.811; 1.461]	0.574
Medical assistant/clinical officer	1.063	[0.741; 1.526]	0.739
Medical officer/specialist	0.849	[0.543; 1.326]	0.471
Allied health workers	0.950	[0.614; 1.472]	0.820
Country			
Malawi	–	–	–
South Africa	2.158	[1.501; 3.103]	<0.001
Tanzania	1.029	[0.725; 1.461]	0.871
Job satisfaction index	0.733	[0.693; 0.776]	<0.001

LR χ^2^=240.9, *p*<0.001, Pseudo R^2^=0.106.

## Discussion

We have used a cross-sectional survey to compare the job satisfaction and intention to leave of different categories of health workers in Tanzania, Malawi, and South Africa. We have found statistically significant differences in job satisfaction and intention to leave between the three countries. The lowest job satisfaction and highest intention to leave were found in South Africa where 47.9% of those surveyed were dissatisfied with their jobs and 41.4% were actively seeking other jobs (Table 4). The differences between countries were confirmed by multiple regression while adjusting for other potential predictors. Differences between health worker cadres were not significant but the multiple regression model analysis did show that health professionals working in public hospitals were less satisfied than those in clinics and health centres, younger health workers were significantly more dissatisfied and more likely to want to quit their jobs, and that lower job satisfaction was significantly associated with intention to leave (Tables 5 and 6).

This article adds to the very small comparative literature on job satisfaction and intention to leave. A few studies have directly compared job satisfaction in different countries. For example, a classic study which compared nursing in different countries, Aiken (44) found that 41.0% of American nurses were dissatisfied with their job, compared to 32.9% in Canada, 37.7% in Scotland, 36.1% in England, and only 17.4% in Germany. Chirwa et al. (25) noted differences in job satisfaction between nurses caring for HIV patients in Lesotho, Malawi, South Africa, Swaziland, and Tanzania. In research comparing nurses working in Malaysia and England, it was found that English nurses were significantly more satisfied with their jobs, although Malaysian nurses showed lower intention to leave (45). Studies comparing doctors from Norway and Germany found significantly higher job satisfaction among Norwegian doctors which was attributed to better working hours, higher salary, and more control over clinical work in Norway (41, 43).

A few comparative studies have explored differences in job satisfaction or intention to leave between different categories of health workers (46–50). Although differences have been noted in these studies they have not always been evaluated statistically. We were not able to confirm significant differences in job satisfaction or intention to leave between different health worker cadres using multiple regression models. Similar findings have been reported elsewhere (66) but a number of authors have shown nurses to be significantly more satisfied with their jobs than doctors (47, 49, 50). Although they did not compare levels of satisfaction, Krogstad et al. (48) demonstrated that the job satisfaction of doctors, nurses and auxiliaries in Norway were influenced by different determinants.

The finding that younger nurses have lower levels of job satisfaction and higher intention to leave has been confirmed in a number of previous studies (5, 44, 67–69) and in a meta-analysis of variables related to job satisfaction (21). Other authors have found lower job satisfaction in public sector health workers in both lower and higher-income countries (27, 31, 40, 70, 71) and the association between job satisfaction and intention to leave has been demonstrated before (14–18).

This article also contributes to the limited research on job satisfaction and intention to leave in the three study countries. South African health professionals had the lowest levels of satisfaction in our study but Chirwa et al. (25) found that nurses from South African and Tanzania had higher mean job satisfaction scores than those from Malawi, Swaziland, and Lesotho. The low levels of job satisfaction among public sector nurses in South Africa has been confirmed in a number of studies (70, 72–74) and a survey of primary health care nurses in rural South Africa also found high turnover intentions as 51.1% planned to leave their current job within 2 years (56). A cross-sectional survey of nurses from Tanzania, Kenya, and Uganda found lower levels of job satisfaction when compared with a European reference group and also that satisfaction was lower among public hospital nurses than those working the private sector (24). In one of the few studies from Malawi, McAuliffe et al. (39) showed that job satisfaction was correlated with elements of organisational justice among a mixed group of mid-level workers.

This survey was a small component of a larger study and suffers from a number of limitations. First, although this is one of the largest health worker job satisfaction surveys to date, the samples were not designed as nationally-representative samples. Resource constraints required regionalised sampling strategies in all three study countries although we were careful to select provinces and regions that were typical. Also, we did not show statistically significant differences between different categories of health workers which may be due to the lower power of these analyses, even though the numbers of respondents in each category were not small. These problems are not unique to this study, since most of the available literature on health worker job satisfaction is based on very small samples of health workers (57). More representative studies will require a significant investment of resources in HRH research in LMICs.

Second, although job satisfaction measurement tools have been validated for use in many different settings (75), local construct validation does not necessarily ensure direct comparability of tools for comparative research. Nevertheless, it is encouraging that measurement equivalence of job satisfaction scales have been demonstrated between different countries (76), and between nurses and doctors (77).

Third, the objective of this study was to measure and compare job satisfaction and intention to leave in the three countries, so we did not collect detailed data on possible determinants, actual working conditions or remuneration. Therefore, the available variables only explained a small proportion of the variation in job satisfaction and intention to leave in the multiple regression models. Also, like similar health worker surveys, we did not investigate if differences in expressed job satisfaction had any real impact on health worker performance or patient care. Further research will be required to properly explain some of the patterns that we have observed and investigate their significance for health service delivery.

Finally, like much of the existing HRH literature, this analysis is based on cross-sectional rather than longitudinal data. As a result, we were not able to measure actual turnover although there is significant empirical evidence linking intention to leave with actual leaving in other settings (78, 79). Cross-sectional studies may also be biased because they only capture the views of health workers that have remained in service. More longitudinal HRH research is an important priority to address these limitations, particularly in LMICs (15, 30).

There are important implications of the findings reported here. This preliminary study demonstrates the need for more detailed comparative HRH research. We have shown differences in the levels of job satisfaction and intention to leave between different groups of health workers from different countries but research is also needed on the relative importance of different determinants of job satisfaction and retention for different health professionals in different contexts. Current HRH interventions are based on fairly standardised generalisations about what is important to health workers but more targeted HRH strategies, based on more differentiated research, may be more important than has been recognised to date.

Better working conditions may not result in higher health worker job satisfaction and retention. We found the lowest levels of satisfaction and highest turnover intentions among South African health professionals which is surprising considering that the health system in South Africa is much better resourced than either Malawi or Tanzania. General living standards are better, per capita public health expenditure is many times higher, and staffing ratios are better (Table 1). The lower satisfaction of health workers in the public sector, compared to the non-governmental or private sector, is also not always attributable to significantly worse resources or remuneration (27, 31, 70). There is a significant affective component to job satisfaction and intention to leave which suggests that the observed differences between countries or sectors are not simply related to differences in job characteristics or working conditions but are influenced by other cultural, economic and political factors such as health worker expectations, organisational culture, labour market conditions, as well as the organisation and militancy of health professionals (5, 80). HRH interventions need to take these dynamics into account since good strategies may be ineffective, or even have contradictory effects, when introduced in a general climate of dissatisfaction. More attention also needs to be given to identifying HR interventions and strategies that improve the general morale and attitudes of health workers.

The observation here, and in other studies, that younger health professionals have lower job satisfaction, and express higher turnover intentions is also cause for concern. There are generational differences between health workers that appear to have less to do with ageing than with significant changes in the underlying motivations, needs, expectations and opportunities of newly qualified health workers. Corresponding changes are required in the selection, training, deployment, and remuneration of health professionals to address the disaffection of young health workers and prevent further deterioration of the HRH crisis.

Suggested interventions to improve job satisfaction have mostly been extrapolated from the organisational factors known to be associated with job satisfaction, so include improving remuneration, workload, physical working conditions, work organisation, supervision, and leadership (5, 23). However, there are very few studies that have rigorously evaluated the impact of such HRH interventions on the job satisfaction of health professionals (81–83), and none from lower income countries. In the absence of such evidence, our results caution against simplistic assumptions about the effectiveness of these interventions. More rigorous intervention research is clearly needed if the existing descriptive studies are to be translated into practical HRH strategies.

## Conclusion

Improving the motivation, performance and retention of health workers are essential steps in addressing the HRH crisis facing LMICs. Job satisfaction is an important determinant of the performance and turnover of health professionals. We have shown differences in the levels of job satisfaction and intention to leave between different groups of health workers from Tanzania, Malawi, and South Africa. Moving from description to intervention requires a better understanding of the different determinants of job satisfaction and intention to leave for different sub-groups of health workers in different countries. Our findings highlight the need for less standardised and more targeted HRH strategies than has been the practice to date.
